# The Novel CFTR Mutation A457P in a Male with a Delayed Diagnosis of Cystic Fibrosis

**DOI:** 10.1155/2011/903910

**Published:** 2011-12-13

**Authors:** Kate H. Cole, Patrick R. Sosnay, Lonny B. Yarmus, Jonathan B. Zuckerman

**Affiliations:** ^1^Division of Pulmonary and Critical Care Medicine, Johns Hopkins University, 1830 East Monument Street, 5th Floor, Baltimore, MD 21208, USA; ^2^Division of Pulmonary and Critical Care Medicine, Maine Medical Center, 22 Bramhall Street, Portland, ME 04102, USA

## Abstract

Cystic fibrosis (CF) is an autosomal recessive disease that may be caused by more than 1000 different mutations in the cystic fibrosis transmembrane conductance regulator (CFTR) gene. We describe the case of a CF patient who was initially diagnosed at 16 years of age after presenting with mild respiratory compromise and pancreatic sufficiency. When genetic testing was first performed using a CF mutation panel, only a single F508del CFTR allele was identified. We subsequently performed testing, which revealed a previously unreported mutation: A457P (p.Ala457Pro, c.1369G>C). The patient's clinical course through adulthood is described, and genotype-phenotype correlation is discussed. The A457P mutation appears to confer a relatively mild phenotype, as is usually observed with CFTR class IV–VI defects. With the advent of more comprehensive and widely available genetic testing techniques, identification of CF genotypes in patients with milder disease variants may help stratify patients for targeted therapy and prevent late complications of the disease.

## 1. Introduction

The median age of diagnosis of cystic fibrosis is 6 months, although it is increasingly common for patients to be diagnosed as adults [1, 2]. The majority of affected patients identified early in life (nearly 90% in the USA) have at least one copy of the F508del mutation, and heterozygotes usually have a second allele that is identified on appropriate mutation panels [3]. However, individuals who are diagnosed later in life are more likely to have mild disease, often associated with CFTR mutations that confer less protein dysfunction and may not be detected on standard, commercially available assays [4, 5]. We describe a patient, diagnosed with CF at age 16 years, who had one F508del mutation and an unidentified allele on standard genetic testing using a CF mutation screening panel. After transfer of care to our institution, a full-sequence CFTR genetic analysis was performed by Ambry Genetics, which revealed an A457P mutation in combination with the F508 deletion. We briefly describe the clinical profile of this patient with the A457P mutation, discuss the established criteria used to diagnose CF, and speculate about the possibility that this newly identified variant is a disease-causing mutation.

## 2. Case Presentation

A 35-year-old Caucasian male presented to the adult cystic fibrosis program at our institution in 2002. He was initially diagnosed with CF at the age of 16 years after evaluation for persistent cough and recurrent bouts of bronchitis. Sweat chloride concentration was 79 mmol/L and 86 mmol/L on subsequent testing. At the time of diagnosis he had never been admitted to the hospital for respiratory infections. He produced about a tablespoon of sputum daily. Sputum cultures frequently grew *Staphylococcus aureus* and *Haemophilus influenza* and occasionally *Pseudomonas aeruginosa*. Utilizing a commercially available CFTR mutation panel, testing at that time showed only a single copy of the F508del mutation while the second allele remained unidentified. Childhood history was notable only for tonsillectomy. He had no problems maintaining weight nor did he suffer from steatorrhea. Both of his parents came from British and Scottish backgrounds. One maternal cousin's daughter was diagnosed with clinical CF. At the time of presentation to our institution, he worked as a teacher at a local university and was completing his doctoral thesis in musicology. As part of his studies, he played wind instruments, and he reported no difficulty with these exercises.

**Figure 1:**
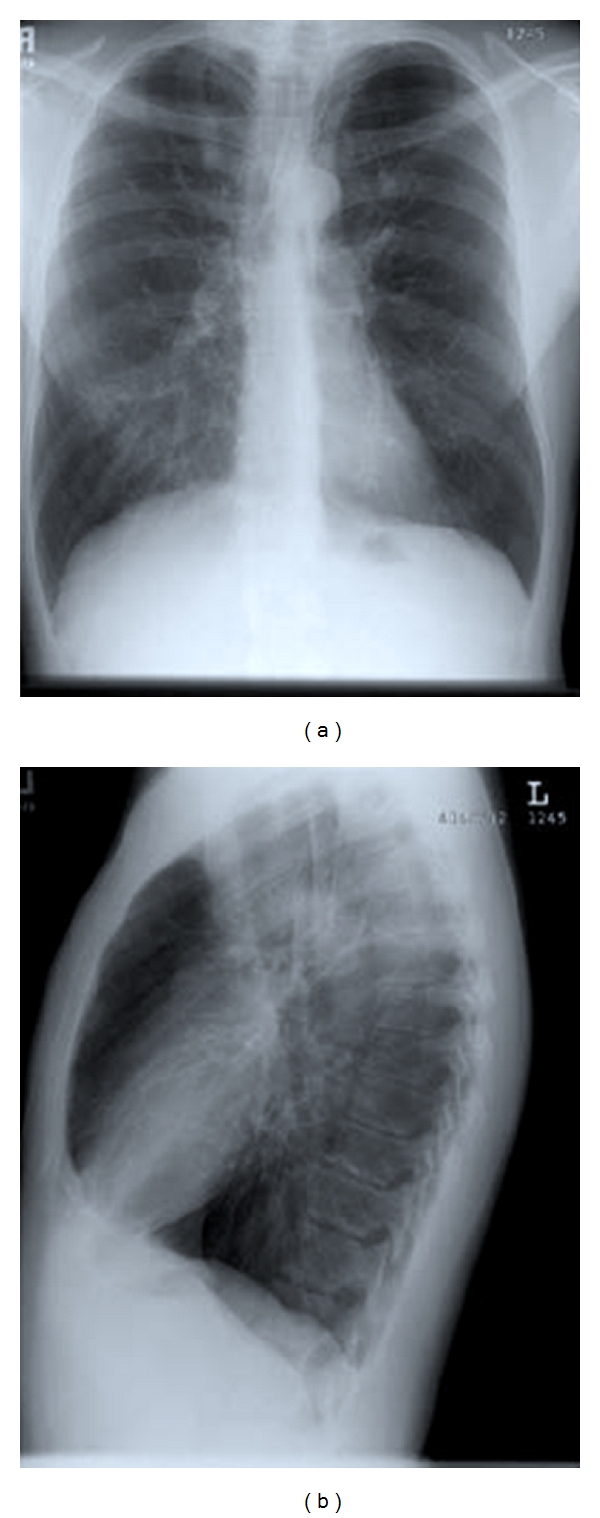
Chest roentgenogram at 35 years of age shows hyperinflation and increased linear markings particularly in the right lower lung zones and perihilar regions corresponding to a Brasfield et al. score of 15 [6].

On examination at our institution, his vital signs were normal. Body mass index was 21.6 kg/m^2^ (104% ideal body weight), and his oxyhemoglobin saturation on room air was 96%. No nasal polyposis or sinus tenderness was present. Pulmonary examination revealed resonance to percussion and symmetric breath sounds with no wheezing or rales. Abdominal exam revealed no organomegaly or areas of tenderness. There was trace digital clubbing. Spirometry showed a moderate obstructive defect: FVC 5.29 L (91% predicted) and FEV1 2.71 L (62% predicted). Chest X-ray demonstrated significant hyperinflation and increased linear markings in the right lower lung zones and perihilar regions (Figure 1). The results of laboratory measurements of liver function, fat-soluble vitamin levels, renal function, glucose and complete blood counts were normal.

## 3. Subsequent Clinical Course

The patient had experienced infertility and requested formal evaluation to ascertain whether having a biological child with his wife was possible. He was referred to a urologist, where physical examination revealed atretic vasa deferentia. The finding was confirmed on transrectal ultrasound, and subsequent semen analysis demonstrated azoospermia. He was aware that he had an unidentified CFTR mutation at the time of presentation to our center. As part of a discussion about family planning, he expressed interest in more comprehensive genetic testing. Full-sequence CFTR analysis was performed by Ambry Genetics. The Ambry Test CF includes a full mutation scan of the CFTR gene by modified temporal temperature gradient electrophoresis analysis followed by dye-terminator DNA sequencing of suspect regions. All CFTR exons as well as relevant intronic regions were amplified using PCR and proprietary primers, essentially as described [8]. Testing revealed an A457P mutation in exon 10 in combination with the F508 deletion. This was the first and only time this variant was observed at Ambry Genetics in over 60,000 chromosomes analyzed [9]. A literature review and examination of the Sick Kids Cystic Fibrosis Mutation database revealed no prior report of the A457P mutation [10]. This mutation is also not seen in the Clinical and Functional Translation of CFTR (CFTR2) database, a locus-specific phenotype-based database of 40,000 CF patients worldwide [11].

**Figure 2:**
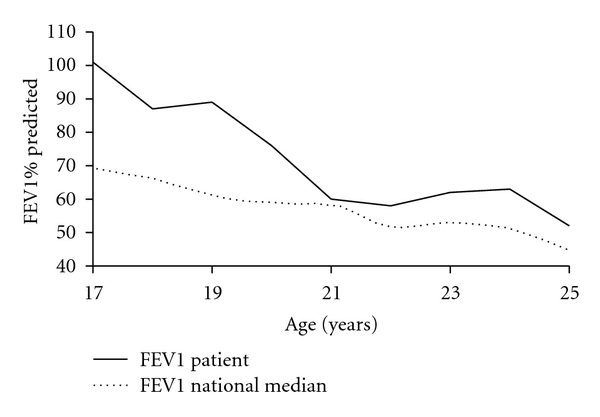
Comparison of lung function of the patient (represented by FEV1% predicted using the Knudson equation) compared to median values reported in CF National Registry for patients in 1990 [7].

The course of his pulmonary function over time was reviewed. Between the ages of 17 and 20 years our patient was actively engaged with a CF center, and his lung function remained above the national median for his age cohort in the USA (Figure 2). However, after age 20 years, he was lost to followup until age 35 years, during which time he experienced accelerated decline in lung function.

## 4. Discussion

Although the diagnosis of CF is usually made during early childhood (70% by age 1 year) [1], late adolescent and adult diagnosis is increasingly common [2]. Adult patients who have a delayed diagnosis often present with atypical features of the disease, and a mild clinical presentation can sometimes delay diagnosis for years. These patients also tend to have unusual genotypes that may not be detectable on routine commercially available mutation panels. Early detection of disease may be of clinical benefit, since many of these patients with mild phenotypes will suffer significant pulmonary decline over time, particularly if left untreated. As seen in our patient, care provided through a CF center may result in long periods of preserved lung function (Figure 2). We speculate that the accelerated decline in his lung function, causing it to approach the national median for CF patients, was due at least in part to a lack of standard therapy during the 15 years that he was lost to followup. Though the diagnosis of CF was made prior to performance of advanced genetic testing in the case of our patient, the clinical course still illustrates the importance of implementing uninterrupted CF care for patients with all presenting CF phenotypes.

The 2008 Cystic Fibrosis Foundation Consensus Report provided recommendations for the diagnostic process to be used for patients who present later in life with clinical features consistent with CF [12]. In addition to these clinical features, there must be some evidence of CFTR dysfunction. That can be determined with functional testing (such as sweat chloride or nasal potential difference measurement) or with genetic testing for CFTR mutations. In the case of our patient, given that he had clinical features (obstructive lung disease with the presence of CF organisms in the sputum and obstructive azoospermia) and sweat chloride evidence of CFTR dysfunction, the diagnosis was established without needing genetic testing.

Analysis of the previously unidentified genetic variation A457P must be considered in the context of its discovery in an individual with an established diagnosis of CF. How do we interpret this newly discovered mutation? According to one schema that has recently gained broad acceptance, mutations in the CFTR gene fall into 1 of 4 categories [3]. They either are (a) known to cause disease, (b) known *not* to cause disease, (c) lie somewhere between these two extremes and cause disease in some patients but not in others (mutations of variable penetrance), or (d) have unknown disease liability (variants of uncertain clinical significance (VUS)). Many of the mutations with variable penetrance (and some that cause CF) have also been seen in patients with single organ system manifestations of CFTR dysfunction but that do not meet the full diagnostic criteria for CF. These have been characterized as “CFTR-related disorders”; however, the lines between a CF-causing mutation and a CFTR-related disease causing mutation are not absolute [3, 4]. Given that CF is an autosomal recessive disease and that our patient is an affected individual, it would appear that A457P is a CF-causing mutation in this instance. Furthermore, this mutation has not been identified in screens of the general “healthy” population done in association with the 1000 Genomes or dbSNP projects [10] and has not been observed in over 1000 individuals undergoing carrier screening suggesting that it is not a common variation [9]. Genetic analysis done by Ambry and other testing companies is proprietary, so the full extent of sequencing is not always known. Therefore, it is possible, although unlikely, that another mutation is present on the A457P chromosome, for example, in an intronic region that may not have been sequenced. When entered into publicly available *in silico* mutation prediction tools PolyPhen [13] and SIFT [14], the A457P mutation, which resides within the nucleotide binding domain 1 of the CFTR, was predicted to be deleterious; however, these methods have not shown adequate sensitivity or specificity in CF to have clinical utility [15]. Taken together, these findings argue for A457P being a disease-causing mutation, though it is still most accurate to formally categorize it as a VUS.

Mutations in the CFTR gene can also be grouped into categories according to the mechanism by which they disrupt CFTR function [4, 16, 17]. Class I mutations result in early truncation of nascent mRNA and generally lead to severe disease in homozygotes (or compound heterozygotes with a Class II mutation). The most common mutation, F508del, results in a misfolded CFTR (Class II) that is targeted for degradation within the endoplasmic reticulum. Homozygotes for this defect again generally develop severe disease. Class III mutations are folded and trafficked to the cell surface but cannot be activated and have no chloride conductance. Mutations in classes IV–VI all have some level of chloride channel function. Individuals who carry Class IV–VI mutations often have milder disease. The relationship between genotype and* pulmonary* phenotype is complex and difficult to predict on an individual basis. However, the relationship between genotype and *pancreatic* phenotype is much stronger, so patients carrying one severe mutation and one limited mutation (Class IV–VI type) are more likely to be pancreatic sufficient [18]. Our patient's presentation of mild respiratory symptoms and pancreatic sufficiency in association with one known Class II mutation suggests that A457P is likely a Class IV–VI mutation (i.e., one that retains some chloride channel function). Functional testing could help elucidate the cellular consequences of the A457P substitution to support the hypothesis that the mutation is deleterious and to assign the mutation to a functional class; however, the considerable resources necessary to perform such confirmatory work would generally make this impractical for a mutation found in a single patient.

## 5. Conclusion

We identified a novel CFTR mutation in a patient who presented with mild pulmonary symptoms and pancreatic sufficiency and who was diagnosed with CF at 16 years of age. Detailed genetic analysis revealed that he carries the novel mutation (A457P) along with the most common CFTR mutation (F508del). The novel mutation appears to be disease causing in this individual but should be regarded as a VUS until more information can be attained about its potential to bring about disease in additional patients. Identification and classification of novel CFTR mutations will become increasingly important as more adult patients with mild clinical presentations and unusual genotypes are being recognized. By continuing to add to the list of known disease-causing mutations, our ability to identify patients with CF will improve and we should be able to provide earlier and more comprehensive treatment and genetic counseling.
